# Viral vector-mediated downregulation of RhoA increases survival and axonal regeneration of retinal ganglion cells

**DOI:** 10.3389/fncel.2014.00273

**Published:** 2014-09-05

**Authors:** Jan Christoph Koch, Lars Tönges, Uwe Michel, Mathias Bähr, Paul Lingor

**Affiliations:** ^1^Department of Neurology, University Medicine GöttingenGöttingen, Germany; ^2^Center for Nanoscale Microscopy and Molecular Physiology of the Brain (CNMPB)Göttingen, Germany

**Keywords:** RhoA, axonal regeneration, retinal ganglion cells, optic nerve crush, axotomy, neuronal survival, RNAi

## Abstract

The Rho/ROCK pathway is a promising therapeutic target in neurodegenerative and neurotraumatic diseases. Pharmacological inhibition of various pathway members has been shown to promote neuronal regeneration and survival. However, because pharmacological inhibitors are inherently limited in their specificity, shRNA-mediated approaches can add more information on the function of each single kinase involved. Thus, we generated adeno-associated viral vectors (AAV) to specifically downregulate Ras homologous member A (RhoA) via shRNA. We found that specific knockdown of RhoA promoted neurite outgrowth of retinal ganglion cells (RGC) grown on the inhibitory substrate chondroitin sulfate proteoglycan (CSPG) as well as neurite regeneration of primary midbrain neurons (PMN) after scratch lesion. In the rat optic nerve crush (ONC) model *in vivo*, downregulation of RhoA significantly enhanced axonal regeneration compared to control. Moreover, survival of RGC transduced with AAV expressing RhoA-shRNA was substantially increased at 2 weeks after optic nerve axotomy. Compared to previous data using pharmacological inhibitors to target RhoA, its upstream regulator Nogo or its main downstream target ROCK, the specific effects of RhoA downregulation shown here were most pronounced in regard to promoting RGC survival but neurite outgrowth and axonal regeneration were also increased significantly. Taken together, we show here that specific knockdown of RhoA substantially increases neuronal survival after optic nerve axotomy and modestly increases neurite outgrowth *in vitro* and axonal regeneration after optic nerve crush.

## Introduction

Neuronal cell death, axonal degeneration and regenerative failure are the central pathological hallmarks of most traumatic and neurodegenerative diseases of the central nervous system (CNS). As these pathomechanisms usually occur concurrently and influence each other, therapeutic approaches should optimally address molecular targets that act on all of these processes. Based on growing evidence, the Rho/ROCK/LIMK-pathway appears to be an excellent candidate to fulfill these criteria (Mueller et al., 2005; Tönges et al., 2011a). However, it is not clear which pathway members are most suitable to be targeted to address specific pathological mechanisms.

The Rho/ROCK/LIMK-pathway is activated by several extracellular growth-inhibitory signals, including Nogo, ephrins (Shamah et al., 2001) and semaphorins (Dontchev and Letourneau, 2003; Lin et al., 2007). Their signaling is mediated through different transmembrane receptors, e.g., the ephrin receptor and the trimeric NogoR/p75/Lingo1 receptor complex. These receptors lead to an activation of Ras homologous member A (RhoA; Wahl et al., 2000), which finally results in growth cone collapse and retraction of neurites (Jalink et al., 1994; Mackay et al., 1995; Tigyi et al., 1996; Katoh et al., 1998; Gu et al., 2013). One of the best-characterized downstream targets of RhoA is the Rho-associated coiled-coil-containing protein kinase (ROCK) that is activated by RhoA (Ishizaki et al., 1997). ROCK itself has several downstream targets that affect both neuronal survival and actin cytoskeleton, including phosphatase and tensin homolog (PTEN), myosin light chain kinase and LIM domain kinase (LIMK; Endo et al., 2003; Tönges et al., 2011a). LIMK phosphorylates and thereby inactivates the actin-depolymerizing factor cofilin which leads to decreased actin turnover and growth cone collapse (Arber et al., 1998). Next to its functions in developmental axon guidance (Luo, 2000), the Rho/ROCK/LIMK-cascade also plays an important role after traumatic lesions of the CNS when it mediates inhibition of axonal regeneration (Moreau-Fauvarque et al., 2003; Mueller et al., 2005). Another downstream target of RhoA is mammalian diaphanous (mDia) which acts on actin and tubulin polymerization independently of ROCK (Watanabe et al., 1997; Luo, 2000).

The involvement of RhoA in retinal ganglion cell (RGC) pathology has been demonstrated previously. Expression levels of RhoA increased significantly in the retina within 7 days after rat optic nerve crush (ONC; Wang et al., 2007) and in the RGC-layer within 8 days after rat optic nerve infarction (Fard et al., 2013). In human glaucoma patients, increased RhoA-protein-levels were observed in the optic nerve head compared to healthy age-matched controls (Goldhagen et al., 2012).

Several groups have assessed the effects of Rho-inhibition *in vitro* and in different animal models (Donovan et al., 1997; Mills et al., 1998; Lehmann et al., 1999; Monnier et al., 2003; Fischer et al., 2004; Zhang et al., 2007; Julien et al., 2008), mostly with positive effects regarding neurite outgrowth and axonal regeneration. To inhibit Rho, all of these studies have used the C3 enzyme from *Clostridium botulinum* that inactivates Rho by ADP-ribosylation (Rubin et al., 1988; Udagawa and McIntyre, 1996). However, C3 is not specific for RhoA, but also modifies the Rho isoforms RhoB and RhoC (Just et al., 2011). Moreover, C3 was shown to activate microglia and trigger the release of nitric oxide and several proinflammatory cyto- and chemokines independently of RhoA/ROCK-activation (Hoffmann et al., 2008). It also acts on astrocytes inducing glial scar formation (Just et al., 2011).

In the present study, we use short hairpin RNA (shRNA) against RhoA expressed by adeno-associated viral vectors (AAV) to specifically analyze the effects of RhoA downregulation in models of neuronal de- and regeneration *in vitro* and *in vivo*. Extending our recently published work on the effects of specific downregulation of ROCK2 and LIMK1 (Koch et al., 2014), we provide important evidence on the functions and therapeutic potential of RhoA, one of their upstream regulators.

## Material and methods

### Cloning, production and testing of AAV

Adeno-associated viral vectors were produced based on the previously published vectors pAAV-9(5)hSyn-DsRed-H1-EGFP-shRNA (= *pAAV-Si-I* (GenBank ID: AY6406334, Michel et al., 2005)) and pAAV-9(5)hSyn-DsRed-H1-RhoA-shRNA3 (Malik et al., 2006). The shRNA sense sequence was AAGCTGACCCTGAAGTTCA for the EGFP-shRNA and CTATGTGGCAGATATTGAA GT for the RhoA-shRNA.

AAV were produced as reported before (Koch et al., 2011a). Briefly, 293-HEK cells were transfected with the specific plasmid mix (for AAV2: pAAV expression vector, pAAV-RC and pHELPER (both from Stratagene, Amsterdam, Netherlands) in a 1:1:1 molar ratio; for AAV2/1: pAAV expression vector, pHELPER, pAAV-RC and pH21 in a 1:1:0.5:0.5 molar ratio) under addition of calcium phosphate and HEPES buffered saline. Cells were harvested 48 h after transfection, and AAV were purified by dialysis and virus gradient centrifugation in iodixanol. To obtain high titer viral stocks, we performed fast protein liquid chromatography (FPLC). Virus stocks were tested on primary cortical neurons for transduction efficacy and toxicity and viral titers were determined using qPCR. Based on previous comparisons of different AAV-serotypes (Koch et al., 2013), we used the serotype AAV2/1 for all *in vitro* experiments whereas all *in vivo* experiments were performed with AAV2.

Efficacy of RhoA downregulation was assessed in rat primary hippocampal cell culture that was prepared as described elsewhere (Audesirk et al., 2001). Neurons were transduced on day *in vitro* (DIV) 1 with AAV in a concentration of 1 × 10^8^ transforming units (TU) per well. On DIV 7, cells were lysed in ice-cold lysis buffer (10 mM Hepes, 142 mM KCl, 5 mM MgCl, 2.1 mM EGTA, IGEPAL, protease and phosphatase inhibitor and dithiothreitol). Cell lysates were sonicated, resolved on SDS-PAGE and blotted on a nitrocellulose membrane. For further standard Western Blot processing, the following antibodies were used: rabbit polyclonal anti-dsRed (Clontech, Heidelberg, Germany), mouse monoclonal anti-RhoA (26C4) (sc-418, Santa Cruz, Heidelberg, Germany) and mouse monoclonal anti-β-tubulin (Sigma, St. Louis, MO, USA). As secondary antibodies, we used horseradish peroxidase-conjugated goat anti-rabbit and anti-mouse antibodies (Santa Cruz). Bands were visualized using enhanced chemiluminescence (ECL-solution: 250 mM Luminol, 90 mM p-coumaric acid, 1 M Tris / HCl, 30% hydrogen peroxide) and quantified with ImageJ software^1^ (open freeware provided by the NIH, Bethesda, MD, USA) (3 independent protein lysates).

### Primary retinal ganglion cells (RGC) and evaluation of neurite outgrowth

Retinal ganglion cells were prepared from 7 days old Wistar rat pups as described before (Koch et al., 2011a). Retinal ganglion cells were enriched to 99.5% purity using a two-step panning protocol for Thy-1 (Barres et al., 1988). Cells were plated in 24-well-plates in a density of 4000 cells per well in RGC medium composed of serum-free neurobasal medium (Gibco, Life Technologies, Darmstadt, Germany) supplemented with sodium-pyruvate (Sigma-Aldrich, Seelze, Germany), glutamine, N-acetyl-cysteine, triiodothyronine, Sato (BSA, transferrin, progesterone, putrescine, sodium selenite; Gibco), forskolin (final concentration 10 mM), human BDNF (final concentration 50 ng/ml; Tebu, Offenbach, Germany), insulin (final concentration 5 mg/ml; Sigma-Aldrich), CNTF (final concentration 10 ng/ml; Tebu) and B27 supplement. Coverslips (Sarstedt, Nümbrecht, Germany) were coated with poly-D-lysine (Sigma-Aldrich) and either laminin (20 μg/ml; Sigma-Aldrich) or chondroitin sulfate proteoglycan (CSPG; 50 μg/ml; major components: neurocan, aggrecan, phosphacan and versican; Chemicon; Darmstadt, Germany).

For viral transduction, the medium was reduced to 250 μl per well at 4 h after seeding and AAV were added in a concentration of 5 × 10^7^ TU per well. After 24 h, 250 μl fresh medium were added to each well. Transduction efficacy of RGCs (>90% after DIV 3) and possible virus toxicity (<10% cell death until DIV 5) were monitored regularly.

To assess neurite outgrowth, micrographs of four randomly chosen visual fields per well of five wells per group were taken on DIV 5 with an inverted microscope (Axiovert, Zeiss, Göttingen, Germany). On the micrographs, the total neurite length per view field was quantified using the neurite tracing module of the ImageJ plugin NeuronJ (Meijering et al., 2004) and then divided by the number of neurons per view field to obtain the mean neurite length per RGC. Results from two independent experiments were statistically evaluated using one-way ANOVA followed by Dunnett’s *post hoc* test with significance at *p* < 0.05.

### Primary midbrain neurons (PMN) and scratch assay

Primary midbrain neurons were prepared from embryonic day 14 Wistar rats as described previously (Lingor et al., 1999). Cells were seeded in a density of 4 × 10^5^ neurons/cm^2^ on poly-L-ornithine/laminin-coated cover slips in PMN-medium (composed of DMEM F12, glucose, BSA, penicillin/streptomycin/neomycin, N1, glutamin and insulin; all Sigma). Four hours after seeding, AAV were added in a concentration of 0.5 × 10^8^ TU per well. On DIV 3, a scratch assay was performed as described before (Tönges et al., 2011b). In brief, a custom-made 2 mm broad silicon rubber scratch device was scratched once across each coverslip. Successful transection of all neurites along the scratch border was verified under a microscope. Following the scratch lesion, half of the medium was exchanged. On DIV 6, the cells were fixed in 4% PFA and a standard immunocytochemistry against tyrosine hydroxylase (TH) was performed (primary antibody: rabbit anti-TH (AB152; Millipore, Darmstadt, Germany); secondary antibody: cy3-labelled mouse anti-rabbit (Dianova, Hamburg, Germany)). Micrographs were taken along both scratch borders with a 20× objective using an Axioplan microscope equipped with a 16-bit grayscale CCD camera and AxioVision 4.6 software (Zeiss). On each micrograph, the lengths of the 10 longest neurites growing across the scratch border were determined using the ImageJ plugin NeuronJ. Per group, 10 micrographs were evaluated. Experiments were repeated in three independent PMN-cultures. Results were statistically evaluated using Student’s *t*-test with significance at *p* < 0.05.

### Animal experiments

All animal experiments were performed according to the regulations of the local animal research council and legislation of the State of Lower Saxony, Germany. Adult female Wistar rats (200–300 g, Charles River, Sulzfeld, Germany) were used for all animal experiments. All surgical procedures (intravitreal virus injection, optic nerve crush, stereotactical injection, axotomy) were performed under deep anesthesia with 10% ketamine (95 mg/kg body weight) and 2% xylazine (7 mg/kg body weight) injected intraperitoneally.

### Intravitreal virus injection, optic nerve crush and GAP43 immunohistochemistry

AAV.EGFP-shRNA or AAV.RhoA-shRNA (1 × 10^9^ TU in 5 μl) were injected intravitreally with a Hamilton syringe as described before (Koch et al., 2011b). An ONC was performed 3 weeks after intravitreal injections as described previously (Koch et al., 2011b). In brief, the orbita was accessed after incision along the orbital rim. After moving the lacrimal gland to the front, the eye bulb was slightly rotated to the side by pulling the superior rectus muscle. Then, the optic nerve was fully exposed by removing the surrounding tissue. A 10-0 polyamide suture (Ethicon, Somerville, NJ, USA) was constricted around the optic nerve for 30 s at a distance of 1.5 mm from its insertion into the eye bulb. Next, the suture was removed and all tissue put back *in situ*. Twenty eight days after ONC, animals were perfused transcardially with 4% PFA and the optic nerve and eye bulb were resected and post-fixed in 4% PFA for 1 h. The retina was flat-mounted in 30% glycerol to assess viral transduction efficacy, which was 20–50% of all RGC. Three animals were excluded from further analysis due to transduction rates <20%. The optic nerves were cryo-protected in 30% sucrose for 48 h and then cryo-sectioned longitudinally (thickness: 16 μm). Cryosections were stained with standard immunohistochemistry methods against GAP43 (monoclonal antibody, Abcam). Cy2-labeled secondary antibodies (Dianova) were used and sections were counter-stained with DAPI (Sigma) and mounted in Moviol (Hoechst). All optic nerve sections were photographed with an Axioplan microscope equipped with AxioVision Software (Zeiss). The number of GAP43 positive axons was counted at defined distances from the crush site. The crush site could be clearly localized due to scar formation and high cell density in the DAPI-stain. For statistical analysis, the numbers of axons at a given distance from the crush site were compared between both groups using the Wilcoxon (Mann-White) test for unpaired data with significance at *p* < 0.05.

### Axotomy of the optic nerve

For axotomy experiments, AAV were injected intravitreally as described above. Three weeks later, the optic nerve was exposed as described above for the ONC and then transsected at 2 mm from the posterior eye pole taking special care not to damage the retinal blood supply. To retrogradely label the RGCs, a 2 mm × 2 mm piece of gel foam (Braun, Melsungen, Germany) was soaked in FluoroGold® (Hydroxystilbamidine; Bio-Trend, Cologne, Germany) and placed on the optic nerve stump. At 14 days after axotomy, the animals were perfused and the retinas flat-mounted as described above. Further examinations were performed under an Axioplan microscope (Zeiss). Using 2.5× and 10× magnifications, the retina was subdivided into transduced and non-transduced areas. For each area-type, four 40× magnification view fields were evaluated at similar distances from the optic disc. Fluorogold®-labeled RGC were counted using a UV filter (365/420 nm). RGC were discriminated from other Fluorogold-positive cells (microglia, macrophages etc.) by morphological criteria (soma size, form of processes). For EGFP-shRNA *n* = 7 animals were evaluated, for RhoA-shRNA *n* = 8 animals were evaluated. Data were evaluated statistically using Wilcoxon (Mann-White) test for unpaired data with significance at *p* < 0.05.

## Results

### Downregulation of RhoA

**Figure 1:**
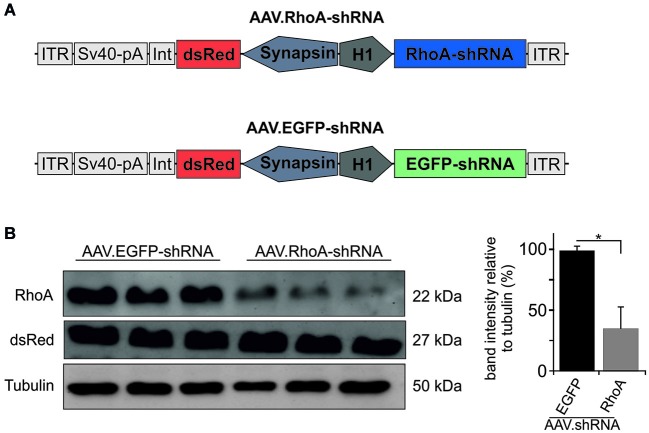
**AAV.shRNA-mediated downregulation of RhoA. (A)** Vector maps of AAV.RhoA-shRNA and the control-vector AAV.EGFP-shRNA. The shRNAs are expressed under the control of an H1 promoter and the fluorophore dsRed is expressed under the control of a human synapsin-1 promoter. ITR: AAV-2 inverted terminal repeat. Int: intron. SV40-pA: SV40 polyadenylation site. **(B)** Immunoblots for RhoA and dsRed of primary hippocampal neurons transduced with AAV.RhoA-shRNA or AAV.EGFP-shRNA (*n* = 3 independent protein lysates per group; bars represent means ± SEM: * *p* < 0.05 according to Student’s *t*-test).

Based on previous testing (Malik et al., 2006), we chose the most potent and specific AAV.shRNA to downregulate RhoA under the control of the H1 promoter and co-expressing the fluorophore dsRed under the control of the human synapsin promoter. As control, we employed the same vector expressing an shRNA against EGFP (Koch et al., 2011a, 2014) (vector maps are shown in Figure 1A). Specific downregulation of the target protein RhoA was confirmed by immunoblot of protein lysates from AAV-transduced primary hippocampal neuron cultures. Transduction with AAV.RhoA-shRNA resulted in a significant downregulation of RhoA expression levels to 35 ± 0.18% compared to AAV.EGFP-shRNA which was set 100% (Figure 1B).

### Inhibition of neurite outgrowth by CSPG is counteracted by downregulation of RhoA

**Figure 2:**
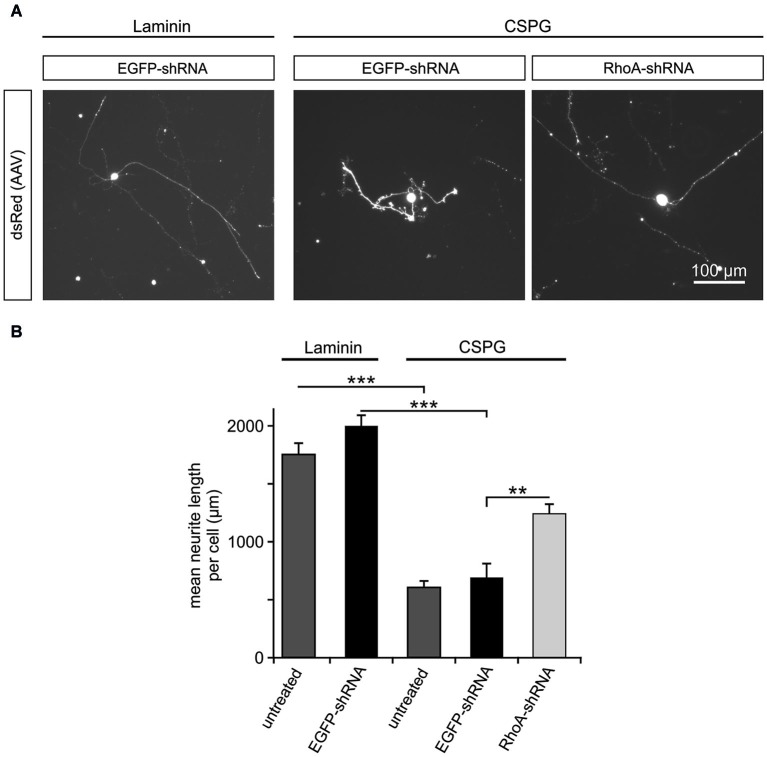
**Downregulation of RhoA partly rescues the inhibitory effects of CSPG on neurite outgrowth in RGC. (A)** Representative micrographs of RGC on DIV 5 without AAV-treatment (untreated) or transduced with AAV.EGFP-shRNA or AAV.RhoA-shRNA (AAV-expressed dsRed). Neurites of the RGC without treatment or transduced with AAV.EGFP-shRNA are clearly shorter on the non-permissive substrate CSPG (on the right side) as compared to laminin (on the left side). In contrast, RGC transduced with AAV.RhoA-shRNA grow long neurites on CSPG. **(B)** Quantification of the mean neurite length per RGC transduced with the given AAV or untreated and plated either on laminin or CSPG (*n* = 2 RGC-cultures; quantification of 20 fields of view per group and experiment; bars represent means ± SEM; ** *p* < 0.005, *** *p* < 0.001, according to one-way ANOVA followed by Dunnett’s *post hoc* test).

RhoA has been reported to be a key intracellular mediator of repulsive signaling from surrounding glia cells on the growing axon (Luo, 2000). To simulate the inhibitory environment present in the adult mammalian CNS, we cultured rat primary RGC on the growth-inhibiting substrate CSPG, which contains some of the key inhibiting molecules produced by glial cells of the CNS *in vivo* (Niederöst et al., 1999). As control, RGC were cultured on the growth permissive coating laminin. On DIV 5, the mean neurite length per neuron was measured for RGC on both coatings with or without viral transduction. There was no significant difference between the untreated RGC (mean neurite length per cell: 1761 ± 92 μm) and RGC transduced with AAV.EGFP-shRNA (1998 ± 101 μm) on laminin. In contrast to RGC cultured on laminin, neurite growth on CSPG-coating was significantly reduced in untreated RGC (mean neurite length 604 ± 56 μm) and RGC transduced with AAV.EGFP-shRNA (689 ± 120 μm). This inhibition of neurite outgrowth was partly rescued by transduction with AAV.RhoA-shRNA, which led to a significantly increased mean neurite length (1240 ± 85 μm) compared to AAV.EGFP-shRNA-treated controls (Figure 2).

### Neurite regeneration after scratch lesion is improved by RhoA-shRNA

**Figure 3:**
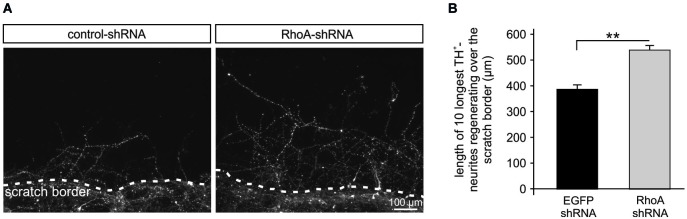
**Rho-shRNA enhances neurite regeneration after mechanical scratch lesion**. **(A)** Representative micrographs of primary midbrain neurons (PMN) transduced with AAV expressing the respective shRNA given on top and immunostained against tyrosine hydroxylase (TH). Photos were taken on DIV 6, 72 h after mechanical scratch lesion. The scratch border is shown at the bottom on each picture with neurites growing across the scratch. **(B)** Quantification showing the mean lengths of the 10 longest TH-positive neurites crossing the scratch border after transduction with AAV expressing the shRNAs given below. Data are given as means ± SEM. ** *P* < 0.005 according to Student’s *t*-test; (*n* = 3 independent PMN-cultures; quantification of 10 × 10 neurites per group per culture, i.e., in total 300 neurites per condition).

Neurites in the CNS exhibit an insufficient regeneration capacity after traumatic or biochemical lesion, e.g., by oxidative stress, which has relevance for the pathogenesis of numerous neurological disorders. To mimic this situation, we performed a scratch assay in rat PMN (Tönges et al., 2011b). Primary midbrain neurons were transduced with AAV.EGFP-shRNA or AAV.RhoA-shRNA and on DIV 3 a mechanical neurite transection was performed using a special rubber device that was scratched across the cover slip 3 days later, cells were fixed and stained against TH to identify dopaminergic neurons. Micrographs were taken along the scratch border and the length of the 10 longest TH-positive neurites growing across the scratch border was assessed. The mean length of these regenerating neurites was significantly increased in the neurons transduced with RhoA-shRNA (538 ± 16 μm) as compared to EGFP-shRNA (387 ± 14 μm; Figure 3).

### Downregulation of RhoA promotes axonal regeneration after optic nerve crush

**Figure 4:**
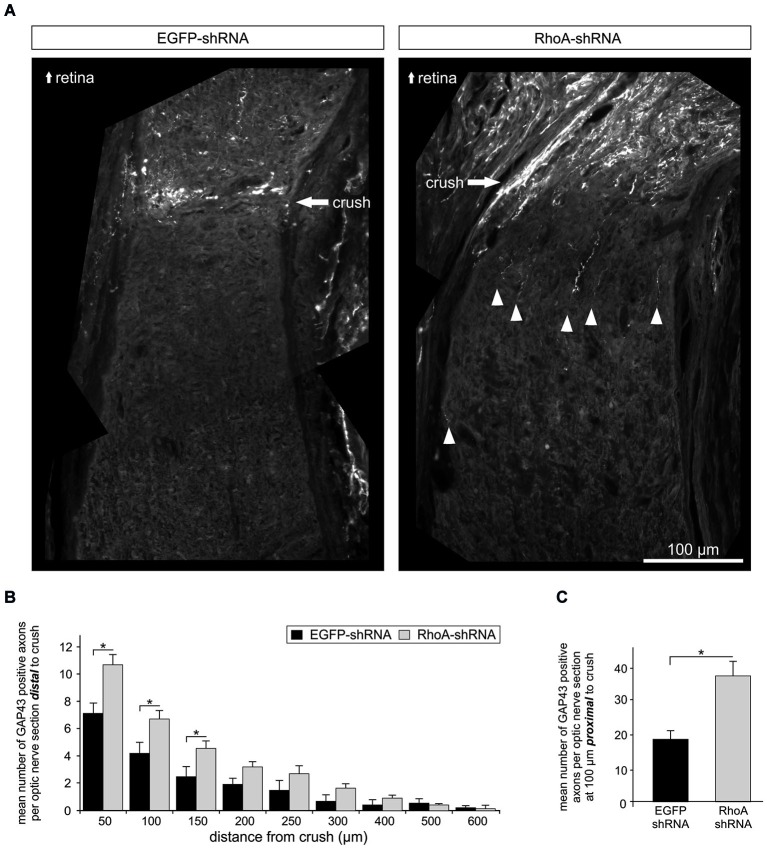
**Knockdown of RhoA increases axonal regeneration after optic nerve crush. (A)** Representative composite photos of optic nerves transduced with AAV.EGFP-shRNA or AAV.RhoA-shRNA and immunostained for GAP43. AAV were injected intravitreally 14 days before crush lesion. Optic nerve sections were obtained 28 days after crush. White arrow-heads point at regenerating (GAP43-positive) axons. **(B)** Quantification of the number of GAP43-positive axons at different distances from the crush site (*n* = 10 optic nerves per group; bars represent means ± SEM; * *p* < 0.05; ** *p* < 0.005 according to Student’s *t*-test). **(C)** Quantification of the number of GAP43-positive axons at 100 μm proximal to the crush site shows a significant increase only for the optic nerves transduced with AAV.RhoA-shRNA (*n* = 10 optic nerves per group; bars represent means ± SEM; * *p* < 0.05, according to Wilcoxon (Mann-White) test for unpaired data).

Next, we analyzed the effects of RhoA downregulation on axonal regeneration *in vivo*. AAV.EGFP-shRNA or AAV.RhoA-shRNA were injected intravitreally in adult female Wistar rats. Three weeks after intravitreal injection, a crush lesion of the optic nerve (ONC) was performed as described before (Koch et al., 2011b). The animals were sacrificed 3 weeks later and the optic nerves were immunostained with an antibody against GAP43 to specifically identify regenerating axons (Schaden et al., 1994). The number of GAP43-positive axons was quantified at increasing distances from the crush site (Figures 4A,B). Transduction with AAV.RhoA-shRNA resulted in a significant increase of regenerating axons that had crossed the lesion site compared to AAV.EGFP-shRNA (*n* = 10 optic nerves per group). This effect was, however, limited to the area close to the crush as no regenerating axons could be observed at distances >600 μm from the crush site in neither group. The number of GAP43-positive axons at 100 μm proximal to the crush site was also increased significantly in the optic nerves transduced with AAV.RhoA-shRNA as compared to AAV.EGFP-shRNA, indicating increased stability of RGC axons following crush lesion (mean number of GAP43-positive neurites per section: EGFP-shRNA: 19 ± 3; RhoA-shRNA: 38 ± 4; Figure 4C).

### RGC survival after optic nerve axotomy is improved by downregulation of RhoA

**Figure 5:**
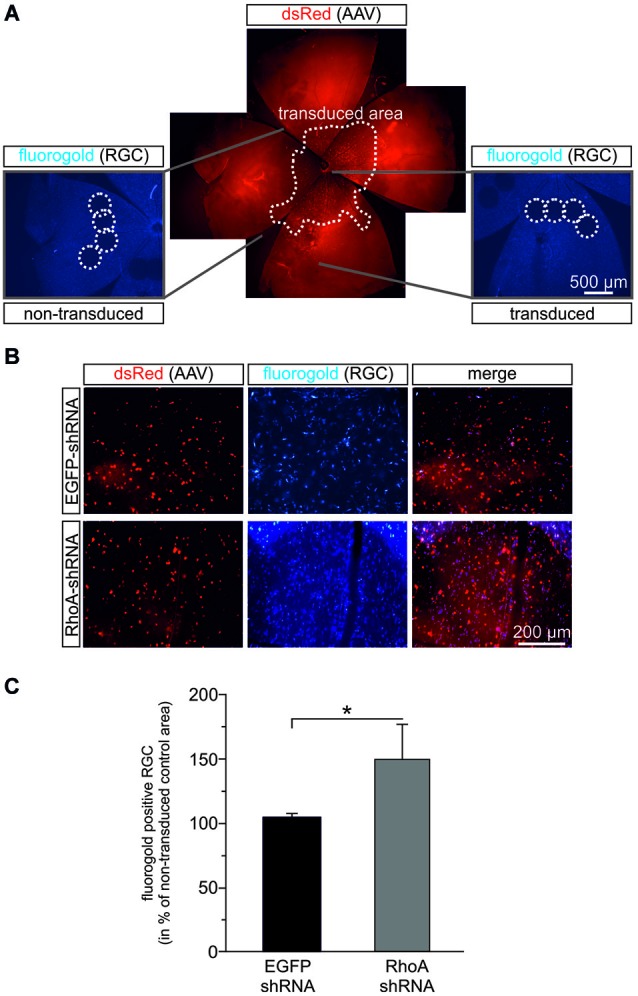
**RhoA-shRNA increases RGC survival after optic nerve axotomy**. **(A)** Composite figure explaining the evaluation method used to assess RGC-survival after optic nerve axotomy. Retina flat mounts were obtained 14 days after transection of the optic nerve. Per retina, four view fields were evaluated in a region that was not transduced by the AAV (dashed circles in the left micrograph) and directly compared to four view fields in an AAV-transduced region (dashed circles on the right micrograph) of the same retina in a similar distance from the optic disc. **(B)** Representative micrographs of transduced view fields from retina flat mounts transduced with the given AAV in red and Fluorogold positive cells in blue. **(C)** Quantification of the number of Fluorogold-positive RGC given in percent of AAV.EGFP-shRNA control (set to 100%) (*n* = 7 retinas for AAV.EGFP-shRNA and *n* = 8 retinas for AAV.RhoA-shRNA; bars represent means ± SEM; * *p* < 0.05 according to Wilcoxon (Mann-White) test for unpaired data).

To test possible pro-survival effects of RhoA-downregulation *in vivo*, we employed the optic nerve axotomy model in adult female Wistar rats. AAV.EGFP-shRNA or AAV.RhoA-shRNA were injected intravitreally. Three weeks later, a complete axotomy of the optic nerve was performed and RG were labeled by application of FluoroGold® to the optic nerve stump. After another 2 weeks, the number of FluoroGold®-labeled RGC was quantified on retinal flat-mounts (Figure 5). Viral transduction rates were similar for both groups: transduced (dsRed-positive) RGC in percent of all (FluoroGold®-positive) RGC: AAV.EGFP-shRNA: 45%, RhoA-shRNA 53% (*p* = 0.534, Student’s *t*-test). To specifically assess effects of the AAV-treatment and to avoid a possible bias caused by differences of RGC numbers between animals and different regions of the retina, we defined four regions per retina that were not transduced by the AAV and directly compared the respective RGC-numbers of these regions with four AAV-transduced regions of the same retina in a similar distance from the optic disc (Figure 5A). Transduction with AAV.RhoA-shRNA resulted in a significantly increased number of RGC (149 ± 27%) compared to AAV.EGFP-shRNA (104 ± 3%; *p* = 0.012, Student’s *t*-test; Figure 5C).

## Discussion

Accumulating evidence suggests that modulation of the Rho/ROCK/LIMK pathway has favorable therapeutic effects in several neurological diseases (Mueller et al., 2005; Tönges et al., 2011a, 2012; Koch et al., 2014). In the present study we analyzed the therapeutic value of the specific knockdown of RhoA in CNS neurons with regards to axonal regeneration and neuronal survival.

We demonstrate that knockdown of RhoA partly rescues CSPG-induced inhibition of neurite outgrowth in RGC *in vitro*, increases neurite regeneration of dopaminergic neurons after scratch lesion *in vitro* and moderately enhances axonal regeneration after ONC *in vivo*. These results extend previous studies employing C3-enzyme in RGC or RhoA-siRNA in other neuronal cells.

In PC12 cells on a permissive substrate, RhoA-shRNA decreased F-actin levels and increased the percentage of neurite-bearing cells, but changes in neurite lengths were not assessed (Fan et al., 2008). RhoA-siRNA abolished semaphorin-3A-induced growth cone collapse in rat dorsal root ganglion cell cultures (Hengst et al., 2006), but no other inhibitory factors were tested in this study. In RGC *in vitro*, Rho-inhibition by C3-enzyme completely rescued inhibition of neurite growth induced by CSPG (Monnier et al., 2003) or myelin-substrates (Lehmann et al., 1999). In contrast to these reported effects of C3-treatment, the Rho-shRNA in our study only partly rescued the growth-inhibiting effects of CSPG. This might be due to the fact that the downregulation reduced intraneuronal RhoA levels only by 65% compared to normal levels resulting in a significantly reduced but possibly still functionally relevant RhoA-activity. Additionally, C3 unspecifically inhibits all Rho-isoforms including RhoA, RhoB and RhoC (Just et al., 2011) in contrast to the target-specific RhoA-shRNA. As RhoB was also shown to modulate actin and tubulin dynamics in neurons (Mackay et al., 1995), it is possible that the other Rho-isoforms mediate growth-inhibiting effects when specifically RhoA is downregulated. In favor of the latter explanation, we recently found that a similar knockdown efficacy (60–70%) of ROCK2 and LIMK1 was sufficient to completely rescue CSPG-induced neurite growth inhibition in RGC (Koch et al., 2014).

It has to be noted that the *in vitro* tests for neurite regeneration after scratch lesion were performed in PMN. Due to the low number of neurons in the RGC-cultures it would have been very difficult to induce a reproducible lesion of the neurites in RGC and to statistically evaluate the effects of RhoA-shRNA. The scratch lesion in PMN on the other hand, is a well-established experimental paradigm (Tönges et al., 2011b). Although it represents a different neuronal cell type, it nicely demonstrates *in vitro* the therapeutic capacity of RhoA-downregulation for neurite regeneration after a traumatic lesion, extending the findings on neurite outgrowth in RGC to another pathological condition and another neuronal cell type. Moreover, these results imply positive effects of RhoA-shRNA in dopaminergic neurons, thus setting the stage for further experiments in models of Parkinson’s disease.

In the ONC model *in vivo*, the pro-regenerative effects of C3-enzyme applied locally in gelfoam (Lehmann et al., 1999) or expressed by intravitreally injected AAV (Fischer et al., 2004) have been examined previously. Both studies reported moderate pro-regenerative effects of the C3-treatment with increased axonal regeneration up to 500 μm distal to the crush lesion, comparable to our present results. A significantly stronger regenerative response could be induced by concurrent C3-treatment and lens injury (Fischer et al., 2004) or by single knockdown of the RhoA downstream targets ROCK2 and LIMK1 (Koch et al., 2014). The most effective therapeutic approaches to foster axonal regeneration in the optic nerve published so far address the PTEN and mTOR pathway (de Lima et al., 2012). Interestingly, there is a putative molecular link between the RhoA-downstream target ROCK2 and PTEN (Tönges et al., 2011a). Comparing the amount and length of regenerating axons of our present study to these previous results, the effects of specific RhoA-downregulation are clearly less pronounced than PTEN and mTOR modulation or C3-treatment and additional lens injury. In line with our results in the optic nerve, RhoA-siRNA applied by intraspinal injections or lumbar puncture had only minor positive effects on motorical outcome in a rat spinal cord injury model although improved sensory function and white matter preservation were reported (Otsuka et al., 2011).

We conclude that the neurite-growth-inhibiting signals *in vivo* employ additional other pathways next to RhoA and that targeting RhoA alone is not sufficient to promote a substantial regenerative response after axonal lesions in the CNS *in vivo*, especially taking into account the more promising previous results employing specific ROCK2- or PTEN-inhibition.

We further report here, that RhoA-shRNA significantly increased survival of RGC after optic nerve axotomy. Several *in vitro* studies in different neuronal cells support the notion that an activation of RhoA results in increased cell death while an inhibition of RhoA has anti-apoptotic effects: Rho-inhibition with C3-enzyme attenuated thrombin-induced cell death in hippocampal neurons (Donovan et al., 1997), inhibited apoptotic membrane blebbing in PC12 cells (Mills et al., 1998), increased survival of organotypic Purkinje cells (Julien et al., 2008) and rescued cortical neurons from phenylalanine-induced apoptosis (Zhang et al., 2007), while overexpression of RhoA caused high levels of apoptosis in cortical neurons during early postnatal development (Sanno et al., 2010). Moreover, it was shown that RhoA is essential for glutamate-induced cell death of cerebellar granule neurons, being activated by elevated intracellular calcium levels and acting via p38α (Semenova et al., 2007). In *Drosophila melanogaster*, the RhoA-homolog Rho1 promoted apoptosis independently of ROCK through its effects on c-Jun NH2-terminal kinase (JNK) signaling (Neisch et al., 2010). On the other hand, a few studies reported negative effects of Rho-inhibition on neuronal survival: C3-enzyme induced apoptosis in different neuroglioma cell lines (Udagawa and McIntyre, 1996) and expression of dominant negative RhoA increased apoptosis of spinal cord motor neurons during embryonal development (Kobayashi et al., 2004). In disease models *in vivo*, there is, to the best of our knowledge, no evidence for the effects of specific RhoA-downregulation on neuronal survival. However, in line with our results, Rho-inhibition with C3-enzyme in the ONC model resulted in an increased RGC-number (Fischer et al., 2004).

Our present data identify the isoform RhoA to be involved in neuronal cell death after optic nerve axotomy *in vivo*. The amount of the pro-survival effect was similar to the effects previously observed for ROCK2-shRNA in the same model system (Koch et al., 2014), suggesting that ROCK2 is the main molecular mediator of the anti-apoptotic effects of RhoA. Due to the limitations of the shRNA-approach discussed above, it is, however, also possible that other known downstream targets of RhoA with anti-apoptotic effects, e.g., JNK or p38 α, are involved. This could strengthen the therapeutic value of RhoA-inhibition compared to ROCK-inhibition with regards to neuronal survival.

Taken together, we show here that knockdown of RhoA resulted in a modest increase of neurite outgrowth of RGC and PMN *in vitro* and of axonal regeneration after ONC *in vivo* while RGC survival after optic nerve axotomy was substantially increased. These findings thus characterize in more detail the specific functions of RhoA with importance to pathobiology of traumatic and degenerative CNS disorders and contribute to an improved understanding of the value of RhoA as a putative therapeutic target.

## Conflict of interest statement

The authors declare that the research was conducted in the absence of any commercial or financial relationships that could be construed as a potential conflict of interest.

## Footnotes

^1^http://imagej.nih.gov/ij/

## Abbreviations

AAV: adeno-associated viral vector
ANOVA: analysis of variance
BSA: bovine serum albumin
CNS: central nervous system
CSPG: chondroitin sulfate proteoglycan
DAPI: 4,6-diamidino-2-phenylindole
DIV: day *in vitro*
GAP43: growth associated protein 43
LIMK: LIM domain kinase
PMN: primary midbrain neurons
ONC: optic nerve crush
PBS: phosphate-buffered saline
PFA: paraformaldehyde
RGC: retinal ganglion cells
RhoA: Ras homologous member A
ROCK: Rho-associated protein kinase
RT-PCR: real-time polymerase chain reaction
siRNA: small interfering RNA
shRNA: small hairpin RNA
TU: transforming units.
